# The impact of grandchild care on depressive symptoms of grandparents in China: The mediating effects of generational support from children

**DOI:** 10.3389/fpubh.2023.1043969

**Published:** 2023-03-20

**Authors:** Yue Hong, Wei Xu, Lijuan Zhao

**Affiliations:** School of Sociology, Wuhan University, Wuhan, China

**Keywords:** grandchild care, depression, financial support, emotional support, instrumental support

## Abstract

**Objectives:**

Despite extensive studies about the direct effect of grandchild care on caregiver depression in China, understanding of its internal influencing mechanism has been limited. After controlling for socioeconomic factors, this study investigated whether the experience of caring for grandchildren had a long-term impact on the depression levels of grandparents, either directly or indirectly through generational support from adult children.

**Methods:**

The subjects of this study were a total of 9,219 adults over 45 who participated in the China Health and Retirement Longitudinal Surveys in 2015 and 2018. We adopted a lag-behind variable to examine the impact of grandchild care on depressive symptoms of grandparents. The proposed mediation model was analyzed using bootstrap modeling, and the KHB method was conducted further to examine differences in the effects of generational support.

**Results:**

The experience of caring for grandchildren had a significant negative correlation with the depression level of Chinese grandparents. Moreover, children's support significantly mediated the impact of parenting experience on grandparents' depression. Significantly, instrumental support mediated the effect to the greatest extent, while emotional support from children contributed the least. The intermediary effect has urban–rural heterogeneity.

**Conclusion:**

These findings indicated that grandchild care significantly inhibited the depression level of Chinese grandparents through increased intergenerational support from adult children. The implications of the study's findings were discussed.

## 1. Introduction

Grandchild care is relatively widespread in China. Approximately 50% of middle-aged and elderly couples care for their grandchildren as families or individuals (1). With increasing life expectancy, more Chinese older adults can take care of their grandchildren (2), sharing time pressure and living costs with adult children's families (3). Chinese grandparents play an increasingly important role in caring for their grandchildren.

However, care activities could be associated with grandparents' mental health. Some studies found that grandchild care could limit grandparents' time and opportunities to care for themselves and bring physical burden and emotional pressure (4), while other studies came to a different conclusion that grandparenting may help the elderly gain respect from family members and derive emotional wellbeing through generational interaction (5, 6). In addition, empirical research results presented transnational differences and the influence of specific cultural contexts on caregivers' mental health. Due to young couples' poverty, AIDS, imprisonment, unmarried pregnancy, unemployment, and illegal drug use, grandparents in the U.S. especially African American grandparents may suffer from being forced to care for their grandchildren (7, 8). However, Chinese grandparents generally do not regard care as a burden (9). Responsibility ethics with Chinese characteristics and intergenerational reciprocity can alleviate grand-parenting pressure (10, 11). For Chinese grandparents, care is a family obligation conducive to intergenerational relations and family unity (12).

Among the elderly in China, ~ 30% of men and 43% of women suffer from depression (13). The existing epidemiological literature shows that depression can reduce physical function, daily living ability, and cognitive ability (14); it is also the most critical factor leading to suicide in the elderly (15). In the context of healthy aging, it is necessary to explore the influencing mechanism of depression outcomes to help grandparents avoid health risks. Researchers have argued that social support plays an important role in improving the mental health of older people (16). Characterized as the care and support that members of society receive from others, social support includes formal support (e.g., government, institutions) and informal support (e.g., family, friends) (17, 18), the latter of which is likely to be affected by grandchild care (19).

Previous research has indicated the protective effects of informal social support in the relationship between caregiving and depression (20, 21), while investigating the mediating effects of different functional aspects of children's support is still lacking, which is particularly important in Chinese traditions of filial piety (22). In fact, receiving financial support from adult children in the form of rewards is associated with a reduction in the psychological stress of raising grandchildren (23). Thus, the present study aimed to explore the effects of caring for grandchildren on the depressive symptoms of Chinese grandparents, as well as the intermediary mechanism of adult children's support. Such efforts can present practical and policy suggestions to manage depression in elderly individuals.

### 1.1. Grandchild care and depressive symptoms

Caring for grandchildren can have positive or negative effects on older adults' depression (24). This controversy may be based on two different points: Role Accumulation Theory and Role Tension Theory. Role Accumulation Theory holds that multiple social roles can enable individuals to achieve social integration and self-satisfaction in different areas of social participation, eventually benefiting mental health (25, 26). With aging, retirement and the reduction of the degree of social integration, it may be more necessary to strengthen the social role than ever before (27). Caring for grandchildren enables grandparents to assume more social roles, which not only meets their intergenerational emotional needs (28) but also promotes their social integration (29) and can bring higher life satisfaction and self-efficacy (30). On the other hand, Role Tension Theory suggests that individuals may face different types of role conflicts when required to perform specific obligations (31). When such role strain exceeds the individual's physical and mental abilities, it causes role pressure, which is harmful to health (32). Providing grandchild care may limit grandparents' time and opportunities to care for themselves (33), maintain personal social activities (34) and assume the role of marriage (8), making them more vulnerable to emotional stress.

### 1.2. Generational support from children as a potential mediator

Social Exchange Theory provides a theoretical possibility for exploring the intermediary role of children's support. According to Social Exchange Theory and Equity Theory, individuals seek equal exchange relationships in interpersonal communication, and unequal exchange may adversely affect mental health (35, 36). It is also applicable in the family field in China.

Grandparenting could strengthen intergenerational relationships and bring rewards from adult children (37). First, based on the principle of reciprocity in intergenerational exchange, providing care for grandchildren reinforces adult children's willingness to return. Second, as a form of social participation, parenting grandchildren makes it more likely for grandparents to receive adult children's support by increasing the intensity and frequency of intergenerational contact (38). Third, sharing the labor cost of parenting also strengthens adult children' capacity to reciprocate in remittances (39). The data of Class2012 confirmed that helping to care for grandchildren can significantly increase the amount of financial support and the frequency of adult children's housework and visits (40).

Financial, emotional and instrumental support have been partially verified as protective factors against depression (41). Due to the acceleration of population migration and the inadequacy of the elderly pension and medical security system, the primary source of livelihood for elderly parents, especially in rural areas of China, comes from their children (42). Additionally, money remittance reflects the return of contribution to adult children, and as a psychological reward, it helps to protect the self-esteem and self-efficacy of the elderly (43).

Chinese adult children are mainly responsible for providing emotional support for the elderly as well (44). Data from China and South Korea support that for parents who do not live together, face-to-face contact and online contact can protect the depression level of elderly parents (45, 46). It also confirms the effectiveness of online contact as a form of emotional contact to protect mental health, especially in the context of China's rapid and extensive urbanization.

Some studies have not verified the mediating effect of instrumental support (19). However, elderly individuals with physical limitations were excluded. Those without physical limitations may experience a sense of incompetence when they are offered instrumental support (47). This may cause sample selection error, and we tried to keep samples with physical limitations. However, grandparents who receive instrumental support from their adult children are associated with poorer physical health and are therefore more likely to provide lower intensity grandchild care than those who are healthier. Considering that those respondents might gain more health benefits with less intensive grandchild care (24), we controlled for respondents' self-rated health status to lessen endogenetic problems.

### 1.3. Differences between urban and rural areas

The literature has mainly discussed the psychological impacts of a reciprocal pattern of behavior. Intergenerational time-for-money exchanges (39) or financial exchange (48) could reduce the depressive symptoms of older grandparents. However, the lack of view of grandparents' support and offspring's feedback as separate but related behaviors hinders further understanding of older grandparents' psychological expectations of generational relationships. We assumed that the result of social transformation brought about by modernization had changed the expectations of the elderly on generational relations. Therefore, the intermediary effect of generational support has urban–rural heterogeneity. Urban areas are characterized by a higher degree of modernization and economic wealth but a stronger emotional connection (49). Moreover, the proportion of urban elderly receiving pension is higher than that of rural elderly (22). Thus, compared with urban residents who are more financially independent, the economic support of adult children of rural residents is a stronger predictor of higher life satisfaction and happiness (41, 50), while emotional support can reduce the risk of depression in urban residents (49). We investigated how parents' expectations of children's support in return would influence whether the mental benefits of grandchild care functioned. This paper adopted a “space for time” analysis strategy, that is, through a comparative study between urban and rural areas, to describe the changes in family support relations in the process of changing from traditional society to modern society (51).

### 1.4. The present study

This study contributes to the literature in three ways. First, we used longitudinal and large-scale national data from China to study the impact of caring for grandchildren on Chinese grandparents' depression and therefore alleviated the deviation caused by the endogeneity of reverse causality to some extent, expanding previous studies that focused on cross-sectional (38) or specific region observation windows (19). Second, we discussed the mechanism of adult children's support in the relationship between grandchild care and caregivers' depression. Previous studies have researched the mediating effect of social participation outside the family (52), as well as the psychological effects of grandparenting, child support, and intergenerational reciprocity patterns (41, 48), and have not adequately explained the context of the relationship between caring for grandchildren and grandparents' depressive symptoms within the family field. In particular, different types of generational support rather than a specific type were incorporated into the mediating model for a more detailed explanation. Finally, by investigating how the mediating effect of children's support changes in different areas of residence, we further explored older people's psychological expectations of intergenerational relationships and the potential realization path of the mental impact of intergenerational exchange. Based on the current evidence, we expect that the depression level of grandparents 2 years later with grandchild care experience will be significantly lower than that of non-caregivers (Hypothesis 1). Additionally, caring for grandchildren will be associated with the level of depression of grandparents 2 years later, directly or indirectly, through changes in economic, emotional and instrumental support from their adult children (Hypothesis 2). Finally, among the three types of generational support, financial support contributes most in rural areas, while emotional support contributes most in urban areas (Hypothesis 3).

## 2. Methods

### 2.1. Data and sample

The data came from the two China Health and Retirement Longitudinal Surveys (CHARLS) hosted by the National Development Research Institute of Peking University. CHARLS adopted probability proportional to size sampling to collect a nationally representative sample of Chinese residents aged 45 and above. CHARLS's follow-up survey covered 450 villages in 150 counties and districts across China. We obtained national tracking survey data in 2015 and 2018 from the official website (http://CHARLS.pseeku.edu.cn). Our analysis was limited to respondents above 45 in 2015 (*n* = 20,085)^1^ who reported having at least one grandchild under 16 at baseline (*n* = 13,949), provided complete answers to the independent variable (*n* = 13,882), demographics and family characteristics (*n* = 13,585), health status (*n* = 12,970), children's support (*n* = 12,806), household per capita expenditure (*n* = 11,073), follow-ups (*n* = 9,944) and at least 8 indicators (53) in the depression level measurement in 2018 (*n* = 9,219).^2^

### 2.2. Measures

#### 2.2.1. Depression

The dependent variable was the level of depression in 2018. According to the epidemiology research center Depression Scale (CESD), respondents were asked to assess their psychological and emotional states within a week. The scale included ten subitems. The 4-point responses were rescaled from little or no (0) to most of the time (3). The total score was between 0 and 30. The higher the score, the more serious the depression. In this study, Cronbach's α was 0.80.

#### 2.2.2. Grandchild care

The independent variable was grandchild care, measured by asking the respondent “Did you spend any time taking care of your grandchildren last year” in the 2015 baseline data. If the respondent answered “Yes”, the variable was assigned 1. If the answer was “No”, the variable was assigned 0. The answer to this question distinguished between “caregivers” and “non-caregivers”.

#### 2.2.3. Generational support from children

Support from children includes financial, emotional, and instrumental support (50, 54, 55) respondents received in the last 12 months. Financial support was measured by the total amount of money and in-kind support respondents received from their children in the past year. We took the logarithm to realize the normal distribution. Because there are no direct measures of emotional intimacy with children in the CHARLS questionnaires, seeing children is considered a form of emotional support (22). In addition, considering the importance of telephone, SMS, and other network contact methods in daily life, we also viewed online contact as an important part of emotional support. Therefore, emotional support was measured by the frequency of respondents' offline meeting and online contact with their children in the past year, and the maximum of the two was taken as emotional support. The 9-point responses were rescaled from almost never (0) to almost every day (8), and the contact frequencies of all children were summed. It should be added that since CHARLS 2015 did not ask these questions to respondents living with their children, the emotional support of these samples was assigned the maximum value of 8. Instrumental support was measured by whether respondents would get help from their children if they needed help with basic daily activities such as eating or dressing at the time of the interview or in the future (56). The answer was a binary variable.

#### 2.2.4. Control variables

The control variables at baseline included gender (female = 0); location (rural = 0); education level (illiterate = 0; primary school and below = 1; secondary school graduation = 2; high school graduation = 3; university and above = 4); marital status (married people living together or in different places due to different jobs were assigned as 0; separation, divorce, widowhood, never married = 1); self-rated health status (poor = 0; general = 1; good = 2); age; whether to live with children (not with children = 0); number of grandchildren under the age of 16; number of surviving children; and household per capita expenditure (logarithmic conversion).

### 2.3. Data analysis

In this study, the Ordinary Least Squares (OLS) model was used to determine whether there was a significant correlation between caring for grandchildren and respondents' depressive symptoms 2 years later. To avoid the endogenous problem caused by mutual cause and effect, the result that the dependent variable lagged behind one period was adopted (57, 58). To study the mediation effects of children's support, we used the bootstrap method to test the direct and indirect effects of generational support from children at baseline, which also reduced potential endogeneity. Since this paper tested several intermediary variables, to explore which intermediary variable contributes the most to the indirect effect, we used the KHB^3^ method (59). Moreover, we performed a robust analysis of the mediating effect grouped according to residence. To take into account the complex sample designs, descriptive statistics used the appropriate design weights provided by the CHARLS teams. Since CHARLS data collected after 2013 only offer cross-sectional weight, weight cannot be used in regression analysis in this study. The outcomes are still acceptable, because there is literature indicating that model analysis is less susceptible to weighting whereas descriptive statistical analysis is more sensitive (60). All statistical analyses were performed with Stata 17.

## 3. Results

### 3.1. Descriptive statistics

The total depression score of Chinese grandparents with grandchildren over 45 years old was 8.513, which is close to mild depression according to the standard (61). Table 1 describes the weighted data and shows that there is a significant correlation between grandchild care and respondents' depressive symptoms 2 years later. In the sample, 58.3% of people (or spouses) provided grandchild care. In demographic and social characteristics, 49% of the samples were male, 68.2% lived in rural areas, more than 90% were married or cohabiting, and the average model education level was primary school or above. The respondents' self-rated health was close to the general level, with a mean age of 60. From family information, 36.5% of respondents lived with their children, and the average number of children and grandchildren was more than 2.

**Table 1:** Descriptive statistics (with weights) and correlation coefficient matrix.

**Variables**	**Mean**	**SD**	**1**	**2**	**3**	**4**	**5**	**6**	**7**	**8**	**9**	**10**	**11**	**12**	**13**	**14**	**15**
1. Depression	8.513	0.179	1														
2. Grandchild care	0.583	0.010	−0.054^***^	1													
3. Gender	0.490	0.004	−0.185^***^	−0.009	1												
4. Location	0.318	0.027	−0.106^***^	0.059^***^	0.004	1											
5. Education	1.284	0.039	−0.205^***^	0.066^***^	0.309^***^	0.252^***^	1										
6. Marry	0.096	0.005	0.087^***^	−0.089^***^	−0.094^***^	0.0120	−0.106^***^	1									
7. Health	0.995	0.015	−0.302^***^	0.035^***^	0.094^***^	0.067^***^	0.119^***^	−0.045^***^	1								
8. Age	60.68	0.197	0.005	−0.156^***^	0.090^***^	0.025^**^	−0.155^***^	0.226^***^	−0.084^***^	1							
9. Co-residence	0.365	0.014	−0.002	0.124^***^	−0.010	0.039^***^	−0.003	0.021^**^	0.004	−0.126^***^	1						
10. Grandchildren	2.490	0.085	0.084^***^	0.067^***^	0.008	−0.158^***^	−0.149^***^	−0.005	−0.062^***^	0.153^***^	−0.006	1					
11. Children	2.686	0.057	0.104^***^	−0.171^***^	−0.019^*^	−0.155^***^	−0.201^***^	0.125^***^	−0.095^***^	0.419^***^	−0.007	0.486^***^	1				
12. Expenditure	8.947	0.042	−0.051^***^	0.042^***^	0.019^*^	0.191^***^	0.178^***^	−0.032^***^	0.038^***^	−0.078^***^	−0.333^***^	−0.097^***^	−0.109^***^	1			
13. Financial support	6.743	0.095	−0.008	0.028^***^	−0.020^*^	−0.056^***^	−0.057^***^	0.017	−0.019^*^	0.126^***^	−0.063^***^	0.123^***^	0.214^***^	0.050^***^	1		
14. Emotional support	18.42	0.354	0.032^***^	−0.038^***^	−0.023^**^	−0.069^***^	−0.119^***^	0.058^***^	−0.021^**^	0.227^***^	0.358^***^	0.360^***^	0.692^***^	−0.175^***^	0.164^***^	1	
15. Instrumental support	0.610	0.012	−0.096^***^	0.059^***^	−0.022^**^	−0.044^***^	−0.024^**^	0.0160	0.100^***^	−0.006	0.091^***^	0.038^***^	0.026^**^	0.004	0.063^***^	0.117^***^	1

*** *p* < 0.01,

** *p* < 0.05,

* *p* < 0.1.

### 3.2. Regression analysis

Table 2 shows the linear regression results of the impact. Model 1 included all control variables and showed that grandchild care reduced grandparents' depression. H1 was verified. Additionally, rural women with a low level of education, poor health, single status at a young age, and having a larger number of grandchildren or children were associated with higher depression levels.

**Table 2:** Regression analysis.

	**Model 1**	**Model 2**	**Model 3**	**Model 4**	**Model 5**	**Model 6**	**Model 7**
**Variables**	**Depression**	**Financial support**	**Depression**	**Emotional support**	**Depression**	**Instrumental support**	**Depression**
	**Coef (SE)**	**Coef (SE)**	**Coef (SE)**	**Coef (SE)**	**Coef (SE)**	**Coef (SE)**	**Coef (SE)**
Gender	−1.482^***^ (−10.91)	−0.137^**^ (−1.98)	−1.491^***^ (−10.98)	−0.291^**^ (−2.31)	−1.495^***^ (−11.01)	−0.025^**^ (−2.34)	−1.509^***^ (−11.15)
Location	−0.648^***^ (−4.06)	−0.275^***^ (−3.39)	−0.666^***^ (−4.17)	0.460^***^ (3.10)	−0.628^***^ (−3.93)	−0.067^***^ (−5.33)	−0.720^***^ (−4.52)
Education	−0.800^***^ (−10.50)	−0.035 (−0.90)	−0.802^***^ (−10.53)	0.131^*^ (1.86)	−0.794^***^ (−10.43)	−0.008 (−1.24)	−0.808^***^ (−10.64)
Marry	1.377^***^ (6.14)	−0.143 (−1.26)	1.367^***^ (6.10)	−0.962^***^ (−4.62)	1.334^***^ (5.94)	0.028 (1.58)	1.407^***^ (6.29)
Health	−2.539^***^ (−27.51)	0.027 (0.57)	−2.537^***^ (−27.50)	0.536^***^ (6.26)	−2.515^***^ (−27.21)	0.077^***^ (10.50)	−2.457^***^ (−26.56)
Age	−0.057^***^ (−6.18)	0.023^***^ (4.89)	−0.055^***^ (−6.01)	−0.008 (−0.93)	−0.057^***^ (−6.22)	0.001 (1.20)	−0.056^***^ (−6.10)
Co–residence	−0.105 (−0.73)	−0.265^***^ (−3.63)	−0.122 (−0.85)	6.850^***^ (51.41)	0.201 (1.23)	0.106^***^ (9.35)	0.009 (0.06)
Grandchildren	0.122^***^ (3.05)	0.021 (1.05)	0.123^***^ (3.08)	0.153^***^ (4.15)	0.128^***^ (3.22)	0.006^**^ (1.98)	0.128^***^ (3.23)
Children	0.233^***^ (3.70)	0.488^***^ (15.27)	0.265^***^ (4.16)	4.913^***^ (84.15)	0.453^***^ (5.41)	0.008 (1.52)	0.241^***^ (3.85)
Expenditure	−0.034 (−0.51)	0.209^***^ (6.28)	−0.020 (−0.30)	0.142^**^ (2.34)	−0.027 (−0.42)	0.024^***^ (4.71)	−0.007 (−0.11)
Grandchild care	−0.442^***^ (−3.31)	0.487^***^ (7.19)	−0.410^***^ (−3.06)	0.467^***^ (3.77)	−0.421^***^ (−3.15)	0.051^***^ (4.85)	−0.387^***^ (−2.90)
Financial support			−0.066^***^ (−3.21)				
Emotional support					−0.045^***^ (−3.98)		
Instrumental support							−1.068^***^ (−8.15)
Constant	15.970^***^ (19.02)	2.142^***^ (5.02)	16.111^***^ (19.17)	0.667 (0.86)	16.000^***^ (19.07)	0.202^***^ (3.03)	16.185^***^ (19.34)
Observations	9,219	9,219	9,219	9,219	9,219	9,219	9,219
*R* ^2^	0.145	0.062	0.146	0.615	0.146	0.029	0.151
*F*	141.96^***^	55.76^***^	131.11^***^	1336.95^***^	131.65^***^	25.09^***^	136.58^***^

*** *p* < 0.01,

** *p* < 0.05,

* *p* < 0.1.

Next, according to the stepwise regression model, the provision of grandchild care positively impacted generational support (Model 2, Model 4, and Model 6). The impact factors of grandchild care decreased after adding generational support from adult children (Model 3, Model 5, and Model 7), which preliminarily showed that generational support could have a mediating effect.

### 3.3. Mediating effect test

This study used the bootstrap method to repeatedly sample 1,000 times to test the mediating effect of adult children's economic, emotional, and instrumental support. It can be seen from Table 3 that the confidence intervals for the indirect effects of children's support all did not include 0, and there were intermediary effects.

**Table 3:** Intermediary effect analysis.

**Mediator effect**	**β (95%CI)**	**SE**	**Z**	***P***
**Financial support**				
Indirect effect	−0.032^***^ (−0.055, −0.014)	0.011	−2.98	0.003
Direct effect	−0.410^***^ (−0.692, −0.146)	0.136	−3.00	0.003
**Emotional support**				
Indirect effect	−0.021^***^ (−0.039, −0.008)	0.008	−2.67	0.008
Direct effect	−0.421^***^ (−0.663, −0.146)	0.130	−3.23	0.001
**Instrumental support**				
Indirect effect	−0.055^***^ (−0.086, −0.032)	0.014	−3.97	0.000
Direct effect	−0.387^***^ (−0.652, −0.118)	0.139	−2.79	0.005

Control variables: Gender, Location, Education, Marital status, Health, Age, Whether parents live with their children, Number of grandchildren, Number of children, Household per capita consumption expenditure.

^*^p < 0.1, ^**^p < 0.05,

*** *p* < 0.01.

**Figure 1:**
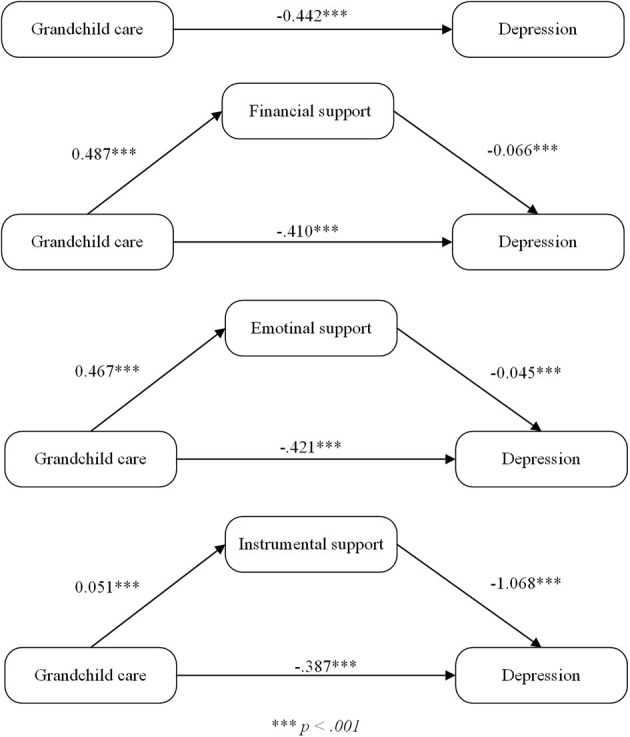
The influence of generational support from children on depression.

Figure 1 illustrates the impact path. When the control variable was included in the model, the negative predictive effect of grandchild care on depressive symptoms was significant (β = −0.442, *p* < 0.001). When economic, emotional and instrumental support from adult children was included in the model as an intermediary variable, grandchild care still had a significant negative predictive effect on depression (β = −0.410, *p* < 0.001; β = −0.421, *p* < 0.001; β = −0.387, *p* < 0.001). Meanwhile, grandchild care had a significant positive effect on adult children's support (β = 0.487, *p* < 0.001; β = 0.467, *p* < 0.001; β = 0.051, *p* < 0.001), and the latter was associated with lower risk of depressive symptoms (β = −0.066, *p* < 0.001; β = −0.045, *p* < 0.001; β = −1.068, *p* < 0.001). H2 was verified.

### 3.4. Comparison of intermediary effects

According to Table 4, generational support mediated 9.38% of the impact. Among them, instrumental support contributed 55.25% to the indirect effect, which was the largest; economic support contributed 27.53%; and emotional support contributed 17.21%.

**Table 4:** A comparative analysis of mediation effect based on the KHB method.

**Mediating variable**	**β**	**SE**	***P*-diff**	***P*-reduced**
Financial support	−0.026	0.011	27.53	5.84
Emotional support	−0.016	0.007	17.21	3.65
Instrumental support	−0.052	0.013	55.25	11.73

The results of grouping regression of urban and rural samples are shown briefly as follows, and the omitted analysis procedure is described in the Supplementary material. Generational support mediated 6.98% in rural samples (*n* = 7,053). Instrumental support contributed 77.07% to the indirect effect, and emotional support contributed 22.93%. The bootstrap model indicated that economic aid was not a mediator. In urban samples (*n* = 2,166), generational support mediated 14.56 percent of grandchild care's depression effect. Instrumental support contributed 32.46 percent; financial support contributed 67.54 percent. Emotional support did not mediate this relationship. H3 was not verified.

## 4. Discussion

This paper suggested that the experience of caring for grandchildren significantly inhibited grandparents' depression using a nationally representative sample. We further tested whether caring for grandchildren weakened the depression level of the grandparents by increasing their adult children's support. Moreover, the data analysis showed that instrumental support mediated the impact to the greatest extent, followed by financial and emotional support. Considering potential differences in the expectations of intergenerational relationships between urban and rural elderly people, this study further compared the intermediary effects of children's support based on residence.

First, caring for grandchildren had a significant negative correlation with the depression level of Chinese grandparents. The results of this study supported the Role Accumulation Theory that caregivers feel they are valuable and experience positive care feelings in intergenerational interaction. It also provided a robust example for discussing the impact of raising grandchildren on caregivers in the Chinese context. In Confucian filial piety culture, grandparents regard caring for grandchildren as a productive role. Similar results have been found in studies of older Chinese Americans and Taiwanese adults under cultural norms that regard care as an obligation; grandparents can give more favorable meaning to the activity of caring for grandchildren and are more willing to care for grandchildren (62, 63). In addition, the role of such cultural norms has also been verified in European countries. In a social and cultural environment where grandparents are expected to provide care, grandparents who do not care for their grandchildren may have negative emotions and experience low life satisfaction (64) and subjective wellbeing (65).

Second, raising grandchildren had a significant positive effect on the economic support, emotional support, and instrumental support from adult children, while the increase in generational support was associated with lower depression levels of grandparents. The research results are consistent with the previous cross-sectional data analysis results (38). On the one hand, the experience of raising grandchildren has a direct protective effect on Chinese grandparents' mental health. The mainstream view is that, out of responsibility and obligation, Chinese parents will raise their offspring (including grandchildren) without any condition (66). On the other hand, the experience of grandchild care can also positively impact the psychological status of grandparents by increasing support from their adult children. Caring for grandchildren can significantly improve the frequency and opportunities for Chinese grandparents to obtain financial support, visit and help with housework (40, 67). In this case, intergenerational resource exchange constitutes a reciprocal state from which the elderly can feel satisfied and happy (68). It can be said that Social Exchange Theory and intergenerational reciprocity are essential mechanisms to explain the mental benefit of grandchild care. It further shows that Social Exchange Theory and the principle of reciprocity also apply to modern Chinese families affected by Confucian culture. As Yan Yunxiang, an anthropologist, observed in the last century, Chinese society, impacted by the new morality prevailing in the market economy, has more intergenerational relations transforming into rational and balanced exchange relations than being unconditionally given by any party (69). It should be noted that this does not mean that grandparents care for grandchildren for the purpose of obtaining adult children's resources. However, the timely feedback of children on care activities, undoubtedly plays an essential role in protecting the mental health of Chinese elderly people to a large extent.

Third, instrumental support mediated the impact of caring for grandchildren on grandparents' depression to the greatest extent, while emotional support from adult children contributed the least. The data from CHARLS2011 and 2013 also support similar results that only the exchange of instrumental support between generations can significantly improve the life satisfaction of grandparents in urban and rural China (22). We also speculated that instrumental support required children to devote higher time and energy costs when compared with offline visits, online contacts, or remittances; thus, it represented a closer intergenerational bond. In addition, a literature review on social support and depression levels also shows that only a small number of research results confirm that children's emotional support is related to the prevention of depression (70). This may be because the primary source of emotional support for the elderly is their spouse, followed by their friends, and finally, their children (70). It should also be noted that, under the current background of large-scale population mobility in China, it is more difficult for elderly parents to have face-to-face contact with their children (45), which may further weaken the emotional dependence of elderly parents on their children. In other words, we can speculate that under the background of modernity, China's intergenerational relations, on the one hand, maintain a certain degree of emotional intimacy and, on the other hand, gradually have the characteristics of rationality and independence.

Finally, urban areas were more inclined to show “time-money” exchange between generations, while rural samples were more inclined to “time–time” exchange of the same type, which was different from what we assumed. The differences between urban and rural areas reflect the transformation in the expectations of the elderly for intergenerational relations in the process of changing from traditional society to modern society. Specifically, the urban elderly put more emphasis on rational logic, while the rural elderly put more emphasis on emotional sense. On the one hand, as the old-age security and social support system in rural areas is more imperfect, elderly people must rely more on their children for daily care in old age (71). On the other hand, caring for grandchild may not raise expectations for monetary compensation because such care usually occurs in three generations of families without financial separation, and people share and coordinate their labor (72). For urban areas, the concept of filial piety has lost its cultural and social foundation, and the popular new morality in the market economy makes the relationship between the two generations more rational and balanced (69).

In conclusion, there are still the following limitations. First, although the inclusion of dependent variables with a lag of one period could alleviate the resulting bias caused by the endogeneity related to reverse causality to a certain extent, we could not confirm whether there were other missing variables in the data that affect the model. Therefore, the results of this study should not be interpreted as a strict causal relationship but only a causal inference under the social science paradigm. Second, in addition to children's support, there might be other relevant factors, such as feelings of care (73), participation in social activities (29), stress, and sleep quality (74), that regulate the relationship between care experience and caregivers. As a result, the data analysis results only reflected the partial impact of care experience. Third, future studies should use more detailed measurements of instrumental support, such as frequency and intensity, and dependent variables, such as care intensities, where data permit. Although we have tried to avoid the selection bias associated with deleting samples with physical limitations occurring in previous literature, there could still be some endogenetic problems. For example, grandparents who received instrumental support provided less intensive care, which resulted in more health benefits for them than those who did not. Fourth, this paper set the intermediary variable as generational support in the baseline data, while generational support in the 2018 data corresponded to a delayed intergenerational reciprocity model. However, Chinese parents' upbringing and adult children's support are not only a kind of immediate feedback model (66). We can try to understand and compare whether there are differences in the impact of immediate and delayed generational support on grandparents' mental health. Fifth, this paper adopted a “space for time” analysis strategy rather than tracking data to explore the influence of modernity on expectations of generational relationships. However, the differences between urban and rural areas also affect the patterns of the family division of labor and the social security system. Therefore, there may be more detailed interpretation work to be done for the conclusions found in this paper. Despite these limitations, our findings still have theoretical and practical significance. Our study supported Role Accumulation Theory and emphasized the protective effect of social integration in the family domain on the mental health of elderly individuals. Specifically, by increasing their adult children's economic support, emotional support and instrumental support, caring for grandchildren achieved intergenerational reciprocity and therefore helped grandparents weaken their negative psychological feelings. Additionally, urban areas were more inclined to “time-money” exchange, while rural samples were more inclined to “time–time” exchange. It may show a trend of rational and independent intergenerational relations in China. With the further deepening of China's aging and the continuous advancement of the active aging process, we can actively advocate for elderly individuals to participate in family care and household activities. Meanwhile, for the elderly who take care of their grandchildren, adult children should pay attention to financial support for grandparents living in urban areas and instrumental support for them in rural areas to protect their psychological state to the greatest extent. Additionally, there is a need for deeper and more comprehensive state pension coverage as well as for affordable, high-quality healthcare, particularly in rural areas where the daily care of the elderly is still largely a family responsibility and grandparents may be compelled to look after their grandchildren in order to guarantee their future care from adult children.

## Data availability statement

Publicly available datasets were analyzed in this study. This data can be found here: http://charls.pku.edu.cn.

## Ethics statement

The CHARLS was approved by the Institutional Review Board at Peking University. Ethics Approval No. IRB00001052-11015. All participants provided informed consent.

## Author contributions

WX and YH designed the study. YH contributed to the collection of literature, data processing, and result analysis and drafted the manuscript. LZ contributed to the data processing, result analysis and review, of the manuscript. All authors approved the current version of this manuscript for publication. All authors contributed to the article and approved the submitted version.

## Footnotes

^1^We limited the sample to people above 45. Due to the intergenerational difference in the age of first marriage, there are sufficiently young grandparents. We believe that the age limit of 45 maximizes the sample utilization rate as much as possible.

^2^Due to missing values in weight variables, the final sample size of descriptive analysis was 9134.

^3^KHB model is a model that calculates the total effect, direct effect and indirect effect by Logit or Probit method. This model was created and developed by Karlson, Holm and Breen. Its main idea is that the total effect can be decomposed into direct effect and indirect effect by direct comparison of coefficients in the linear model.

## References

[1] Zhou P. Supporter characteristics of providing grandchild care. Soc Sci of Bj. (2020) 203:90–101. Available online at: https://kns.cnki.net/kcms2/article/abstract?v=3uoqIhG8C44YLTlOAiTRKibYlV5Vjs7i8oRR1PAr7RxjuAJk4dHXou4iA1UKkwF3fRLIUVg6MBsxLm-bDmUeWefGb989NFAD&uniplatform=NZKPT

[2] Zhou MZ Kan MY He GY. Intergenerational co-residence and young couple's time use in China. Chin Sociol Rev. (2021) 54:401–31. 10.1080/21620555.2021.1972285

[3] Chen JM Chen Q. Parent-child socioeconomic statuses and co-residence: an analysis of living arrangements in China. Sociol Stud. (2016) 31:73–97. Available online at: https://kns.cnki.net/kcms2/article/abstract?v=3uoqIhG8C44YLTlOAiTRKibYlV5Vjs7ijP0rjQD-AVm8oHBO0FTadktOmXKla-DDBAlpLiXPaK5RFnlw4-0C8Nk2ZqU7_bLj&uniplatform=NZKPT

[4] Musil CM Gordon NL Warner CB Zauszniewski JA Standing T Wykle M. Grandmothers and caregiving to grandchildren: continuity, change, and outcomes over 24 months. Gerontologist. (2011) 51:86–100. 10.1093/geront/gnq061 20724656 PMC3018867

[5] Tang FY Li K Jang H Rauktis MB. Depressive symptoms in the context of Chinese grandparents caring for grandchildren. Aging Ment Health. (2021) 26:1120–6. 10.1080/13607863.2021.1910788 33843385

[6] Zeng Y Chen YC Lum T. Longitudinal impacts of grandparent caregiving on cognitive, mental, and physical health in China. Aging Ment Health. (2021) 25:2053–60. 10.1080/13607863.2020.1856779 33291945

[7] Blustein J Chan S Guanais FC. Elevated depressive symptoms among caregiving grandparents. Health Serv Res. (2004) 39:1671. 10.1111/j.1475-6773.2004.00312.x 15533181 PMC1361092

[8] Conway F Jones S Speakes-Lewis A. Emotional strain in caregiving among African American grandmothers raising their grandchildren. J Women Aging. (2011) 23:113–28. 10.1080/08952841.2011.561142 21534103

[9] Tang FY Xu L Chi I Dong XQ. Psychological well-being of older Chinese-American grandparents caring for grandchildren. J Am Geriatr Soc. (2016) 64:2356–61. 10.1111/jgs.14455 27641829 PMC5118144

[10] Lai D. Filial piety, caregiving appraisal, and caregiving burden. Res Aging. (2010) 32:200–23. 10.1177/016402750935147528201932

[11] Miyawaki CE. A review of ethnicity, culture, and acculturation among Asian caregivers of older adults (2000-2012). Sage Open. (2015) 5:1–5. 10.1177/2158244014566365 26229736 PMC4517686

[12] Chen F Liu GY Mair CA. Intergenerational ties in context: grandparents caring for grandchildren in China. Soc Forces. (2011) 90:571–94. 10.1093/sf/sor012 22544978 PMC3337769

[13] Lei XY Sun XT Strauss J Zhang P Zhao YH. Depressive symptoms and ses among the mid-aged and elderly in China: evidence from the China Health and Retirement Longitudinal Study national baseline. Soc Sci Med. (2014) 120:224–32. 10.1016/j.socscimed.2014.09.028 25261616 PMC4337774

[14] de Paula JJ Diniz BS Bicalho MA Albuquerque MR Nicolato R de Moraes EN. Specific cognitive functions and depressive symptoms as predictors of activities of daily living in older adults with heterogeneous cognitive backgrounds. Front Aging Neurosci. (2015) 7:139. 10.3389/fnagi.2015.00139 26257644 PMC4507055

[15] Liebetrau M Steen B Skoog I. Depression as a risk factor for the incidence of first-ever stroke in 85-year-olds. Stroke. (2008) 39:1960–5. 10.1161/STROKEAHA.107.490797 18451342

[16] Bai YL Bian F Zhang LX Cao YM. The impact of social support on the health of the rural elderly in China. Int J Environ Res Public Health. (2020) 17:2004. 10.3390/ijerph17062004 32197415 PMC7143868

[17] Joseph S. Social support - an interactional-view - Sarason, Br, Sarason, Ig, Pierce, Gr. Behav Res Ther. (1992) 30:82–3. 10.1016/0005-7967(92)90110-3

[18] Chi Z Han H. Urban-rural differences: the impact of social support on the use of multiple healthcare services for older people. Front Public Health. (2022) 10:851616. 10.3389/fpubh.2022.851616 35493353 PMC9051021

[19] Zhou J Mao WY Lee Y Chi I. The impact of caring for grandchildren on grandparents' physical health outcomes: the role of intergenerational support. Res Aging. (2017) 39:612–34. 10.1177/0164027515623332 26733495

[20] Whitley DM Kelley SJ Lamis DA. Depression, social support, and mental health: a longitudinal mediation analysis in African American custodial grandmothers. Int J Aging Hum Dev. (2016) 82:166–87. 10.1177/0091415015626550 26798077

[21] Hayslip B Blumenthal H Garner A. Social support and grandparent caregiver health: one-year longitudinal findings for grandparents raising their grandchildren. J Gerontol B Psychol Sci Soc Sci. (2015) 70:804–12. 10.1093/geronb/gbu165 25477430

[22] Wu FY. Intergenerational support and life satisfaction of older parents in China: a rural-urban divide. Soc Indic Res. (2021) 160:1071–98. 10.1007/s11205-021-02672-0

[23] Kim J Park EC Choi Y Lee H Lee SG. The impact of intensive grandchild care on depressive symptoms among older Koreans. Int J Geriatr Psychiatry. (2017) 32:1381–91. 10.1002/gps.4625 27905151

[24] Di Gessa G Glaser K Tinker A. The health impact of intensive and nonintensive grandchild care in Europe: new evidence from SHARE. J Gerontol B Psychol Sci Soc Sci. (2016) 71:867–79. 10.1093/geronb/gbv055 26315046 PMC4982385

[25] Sieber SD. Toward a theory of role accumulation. Am Sociol Rev. (1974) 39:567–78. 10.2307/2094422

[26] Zhao DT Zhou ZL Shen C Ibrahim S Zhao YX Cao D. Gender differences in depressive symptoms of rural Chinese grandparents caring for grandchildren. BMC Public Health. (2021) 21:1–17. 10.1186/s12889-021-11886-3 34635088 PMC8507248

[27] Carstensen LL. Evidence for a life-span theory of socioemotional selectivity. Curr Dir Psychol Sci. (1995) 4:151–6. 10.1111/1467-8721.ep11512261

[28] Mahne K Huxhold O. Grandparenthood and subjective well-being: moderating effects of educational level. J Gerontol B Psychol Sci Soc Sci. (2015) 70:782–92. 10.1093/geronb/gbu147 25324294

[29] Yang X Yin DD. The protective effect of caring for grandchildren on the mental health of the elderly: a structural equation modeling analysis. Int J Environ Res Public Health. (2022) 19:1255. 10.3390/ijerph19031255 35162285 PMC8834749

[30] Choi S Zhang ZM. Caring as curing: grandparenting and depressive symptoms in China. Soc Sci Med. (2021) 289:114452. 10.1016/j.socscimed.2021.114452 34624620

[31] Goode WJ A. theory of role strain. Am Sociol Rev. (1960) 25:483–96. 10.2307/2092933

[32] Pearlin LI. The sociological-study of stress. J Health Soc Behav. (1989) 30:241–56. 10.2307/2136956 2674272

[33] Baker LA Silverstein M. Preventive health behaviors among grandmothers raising grandchildren. J Gerontol Ser B. (2008) 63:S304–11. 10.1093/geronb/63.5.S304 18818451 PMC2633920

[34] Xu HW. Physical and mental health of Chinese grandparents caring for grandchildren and great-grandparents. Soc Sci Med. (2019) 229:106–16. 10.1016/j.socscimed.2018.05.047 29866373 PMC6261790

[35] Dowd JJ. Aging as exchange: a preface to theory. J Gerontol. (1975) 30:584. 10.1093/geronj/30.5.584 1181364

[36] Ingersolldayton B Antonucci TC. Reciprocal and nonreciprocal social support: contrasting sides of intimate-relationships. J Gerontol. (1988) 43:S65–73. 10.1093/geronj/43.3.S65 3361097

[37] Kim JH. Grandparenting, filial piety, and well-being of Chinese-American older adults. Curr Sociol. (2021) 70:860–79. 10.1177/0011392121105922127236539

[38] Tang SL Yang TL Ye CY Liu MX Gong Y Yao L. Research on grandchild care and depression of Chinese older adults based on CHARLS2018: the mediating role of intergenerational support from children. BMC Public Health. (2022) 22:1–14. 10.1186/s12889-022-12553-x 35045856 PMC8772115

[39] Cong Z Silverstein M. Intergenerational time-for-money exchanges in rural China: does reciprocity reduce depressive symptoms of older grandparents? Res Hum Dev. (2008) 5:6–25. 10.1080/15427600701853749

[40] Xu Q. More than upbringing: parents' support and effect on filial duty. Chin J Sociol. (2017) 37:216–40. 10.15709/hswr.2017.37.1.216

[41] Yang Y Evandrou M Vlachantoni A. The impact of living arrangements and intergenerational support on the health status of older people in China: are rural residents disadvantaged compared to urban residents? Ageing Soc. (2021) 43:1–26. 10.1017/S0144686X21000702

[42] Wu XY Li LX. The motives of intergenerational transfer to the elderly parents in China: consequences of high medical expenditure. Health Econ. (2014) 23:631–52. 10.1002/hec.2943 23681718 PMC4160912

[43] Kim KM Lee JJ Chung US. Perceived health status of and moderating factors in elderly people caring for their grandchildren. Psychiatry Investig. (2020) 17:275. 10.30773/pi.2019.0115 32252514 PMC7176558

[44] Tang Y. Obligation of Filial Piety, Adult Child Caregiver Burden, Received Social Support, and Psychological Wellbeing of Adult Child Caregivers for Frail Elderly People in Guangzhou (2006).

[45] Jia YH Ye ZH. Impress of intergenerational emotional support on the depression in non-cohabiting parents. World J Clin Cases. (2019) 7:3407–18. 10.12998/wjcc.v7.i21.3407 31750325 PMC6854408

[46] Roh HW Lee Y Lee KS Chang KJ Kim J Lee SJ. Frequency of contact with non-cohabitating adult children and risk of depression in elderly: a community-based three-year longitudinal study in Korea. Arch Gerontol Geriatr. (2015) 60:183–9. 10.1016/j.archger.2014.09.007 25442783

[47] Choi K Jeon GS Jang KS. Gender differences in the impact of intergenerational support on depressive symptoms among older adults in Korea. Int J Environ Res Public Health. (2020) 17:4380. 10.3390/ijerph17124380 32570826 PMC7344536

[48] Lee HJ Lyu J Lee CM Burr JA. Intergenerational financial exchange and the psychological well-being of older adults in the republic of Korea. Aging Ment Health. (2014) 18:30–9. 10.1080/13607863.2013.784955 23581289

[49] Wang CX Liu ZK Chen TY Wang JF Zhang X Han BX. Intergenerational support and depressive symptoms in old age: the difference between urban and rural China. Front Public Health. (2022) 10:1007408. 10.3389/fpubh.2022.1007408 36466487 PMC9709321

[50] Silverstein M Cong Z Li SZ. Intergenerational transfers and living arrangements of older people in rural China: consequences for psychological well-being. J Gerontol B Psychol Sci Soc Sci. (2006) 61:S256–66. 10.1093/geronb/61.5.S256 16960239

[51] Wu XG Xie Y. Does the market pay off? Earnings returns to education in urban China. Am Sociol Rev. (2003) 68:425–42. 10.2307/1519731

[52] Zhao XY Liu HY Fang BY Zhang Q Ding H Li TY. Continuous participation in social activities as a protective factor against depressive symptoms among older adults who started high-intensity spousal caregiving: findings from the China Health and Retirement Longitudinal Survey. Aging Ment Health. (2021) 25:1821–9. 10.1080/13607863.2020.1822283 32954798

[53] Liang Y. Heterogeneity in the trajectories of depressive symptoms among elderly adults in rural China: the role of housing characteristics. Health Place. (2020) 66:102449. 10.1016/j.healthplace.2020.102449 33011488

[54] Hoff A. Patterns of intergenerational support in grandparent-grandchild and parent-child relationships in Germany. Ageing Soc. (2007) 27:643–65. 10.1017/S0144686X07006095

[55] Tang SL Yao L Li ZJ Yang TL Liu MX Gong Y. How do intergenerational economic support, emotional support and multimorbidity affect the catastrophic health expenditures of middle-aged and elderly families? Evidence from CHARLS2018. Front Public Health. (2022) 10:828. 10.3389/fpubh.2022.872974 35462809 PMC9024169

[56] Zheng RR Yu MY Huang L Wang F Gao BZ Fu DD. Effect of intergenerational exchange patterns and intergenerational relationship quality on depressive symptoms in the elderly: an empirical study on CHARLS data. Front Public Health. (2022) 10:3624. 10.3389/fpubh.2022.1009781 36262237 PMC9574018

[57] Yang YC Boen C Gerken K Li T Schorpp K Harris KM. Social relationships and physiological determinants of longevity across the human life span. Proc Natl Acad Sci U S a. (2016) 113:578–83. 10.1073/pnas.1511085112 26729882 PMC4725506

[58] Thomas PA. Gender, social engagement, and limitations in late life. Soc Sci Med. (2011) 73:1428–35. 10.1016/j.socscimed.2011.07.035 21906863

[59] Kohler U Karlson KB Holm A. Comparing coefficients of nested nonlinear probability models. Stata J. (2011) 11:420–38. 10.1177/1536867X1101100306

[60] Chambers RL Skinner CJ. Analysis of Survey Data. New York, NY: Wiley (2003). 10.1002/0470867205

[61] Boey KW. Cross-validation of a short form of the CES-D in Chinese elderly. Int J Geriatr Psychiatry. (1999) 14:608–17. 10489651 10.1002/(sici)1099-1166(199908)14:8<608::aid-gps991>3.0.co;2-z

[62] Xu L Tang FY Li LW Dong XQ. Grandparent caregiving and psychological well-being among Chinese American older adults-the roles of caregiving burden and pressure. J Gerontol a Biol Sci Med Sci. (2017) 72:S56–62. 10.1093/gerona/glw186 28575256

[63] Ku L Stearns SC Van Houtven CH Lee S Dilworth-Anderson P Konrad TR. Impact of caring for grandchildren on the health of grandparents in Taiwan. J Gerontol B Psychol Sci Soc Sci. (2013) 68:1009–21. 10.1093/geronb/gbt090 24056691

[64] Arpino B Bordone V Balbo N. Grandparenting, education and subjective well-being of older Europeans. Eur J Ageing. (2018) 15:251–63. 10.1007/s10433-018-0467-2 30310372 PMC6156724

[65] Neuberger FS Haberkern K. Structured ambivalence in grandchild care and the quality of life among European grandparents. Eur J Ageing. (2014) 11:171–81. 10.1007/s10433-013-0294-4 28804324 PMC5549145

[66] Fei XT. The problem of supporting the aged in the change of family structure: on the change of Chinese family structure. J Pek Uni. (1983) 7–16. Available online at: https://kns.cnki.net/kcms2/article/abstract?v=3uoqIhG8C44YLTlOAiTRKth5mPLKqXjbyzE23kHsboMTrVKzB-E6C66uKWk-ennEWOtlVVdvMGMjO9pEIZDUPdjwgk2EAn5u&uniplatform=NZKPT

[67] Lei L. Sons, daughters, and intergenerational support in China. Chin Sociol Rev. (2013) 45:26–52. 10.2753/CSA2162-0555450302

[68] Xu Q Wang JS. Influence of intergenerational reciprocity on the satisfaction of the Chinese senior citizens. J Southeast Univ. (2019) 21:104–15. Available online at: https://kns.cnki.net/kcms2/article/abstract?v=3uoqIhG8C44YLTlOAiTRKibYlV5Vjs7iLik5jEcCI09uHa3oBxtWoIuIDy5Q-15liw-RYHd3ZBSHsgviQWDlBHjONPuQsuxJ&uniplatform=NZKPT

[69] Yan Y. Private Life Under Socialism: Love, Intimacy, and Family Change in a Chinese Village, 1949–1999. (2003). p. 214. 10.1515/9780804764117

[70] Gariepy G Honkaniemi H Quesnel-Vallee A. Social support and protection from depression: systematic review of current findings in western countries. Br J Psychiatry. (2016) 209:286–95. 10.1192/bjp.bp.115.169094 27445355

[71] Liu CB Gong NX. The influence of intergenerational caring on residence intention of old floating people. Chin J Pop Sci. (2020) 196:102–12. Available online at: https://kns.cnki.net/kcms2/article/abstract?v=3uoqIhG8C44YLTlOAiTRKibYlV5Vjs7i8oRR1PAr7RxjuAJk4dHXoqmsJ0NR0BTUmB7uTtOp64wMiWDe8iRn7bK27XM5ehhI&uniplatform=NZKPT

[72] Leung JCB. Family support for the elderly in China. J Aging Soc Policy. (1997) 9:87–101. 10.1300/J031v09n03_05 10186888

[73] Smith GC Lee J. Appraisals of self in the caregiver role as made by married custodial grandparents. Fam Relat. (2021) 70:179–94. 10.1111/fare.12451 33424072 PMC7787393

[74] Li SJ Xu HL Li YL. Influence of grandparenting stress, sleep quality, and grandparenting type on depressive symptoms among Chinese older adults who care for their grandchildren: a moderated-mediation study. Curr Psychol. (2021) 1–11. 10.1007/s12144-021-01787-4

