# Supplementary material for: The impact of grandchild care on depressive symptoms of grandparents in China: The mediating effects of generational support from children
Source: Front Public Health. 2023 Mar 20;11:1043969. doi: 10.3389/fpubh.2023.1043969 (PMC10067760; doi:10.3389/fpubh.2023.1043969)
Supplement: Supplementary file 1 [file Data_Sheet_1.docx]

Supplementary Material

# Supplementary Tables

**Figure S1.** Sample screening process.

**Table S1.** Descriptive statistics of urban and rural areas (with weights).

| Variables | Urban (N = 2,138) | | Rural (N = 6,996) | |
| --- | --- | --- | --- | --- |
|  | Mean | SD | Mean | SD |
| Depression | 7.429 | 0.252 | 9.019 | 0.182 |
| Grandchild care | 0.633 | 0.016 | 0.559 | 0.011 |
| Gender | 0.493 | 0.008 | 0.488 | 0.004 |
| Education | 1.729 | 0.054 | 1.076 | 0.027 |
| Marry | 0.101 | 0.011 | 0.094 | 0.005 |
| Health | 1.069 | 0.021 | 0.960 | 0.016 |
| Age | 61.700 | 0.378 | 60.200 | 0.197 |
| Co-residence | 0.373 | 0.024 | 0.362 | 0.017 |
| Grandchildren | 1.918 | 0.068 | 2.757 | 0.097 |
| Children | 2.305 | 0.068 | 2.864 | 0.058 |
| Expenditure | 9.366 | 0.075 | 8.752 | 0.027 |
| Financial support | 6.491 | 0.147 | 6.861 | 0.104 |
| Emotional support | 16.910 | 0.521 | 19.120 | 0.367 |
| Instrumental support | 0.562 | 0.026 | 0.632 | 0.013 |

**Table S2.** Regression analysis of urban and rural areas.

|  | Urban (N = 2,166) | Rural (N = 7,053) |
| --- | --- | --- |
| Variables | Depression | Depression |
|  | Coef (SE) | Coef (SE) |
| Gender | -1.159***(-4.56) | -1.612***(-10.00) |
| Education | -0.702***(-5.40) | -0.794***(-8.40) |
| Marry | 0.207(0.49) | 1.772***(6.74) |
| Health | -2.483***(-13.38) | -2.568***(-24.16) |
| Age | -0.078***(-4.56) | -0.047***(-4.29) |
| Co-residence | -0.335(-1.20) | -0.034(-0.21) |
| Grandchildren | 0.224**(2.29) | 0.107**(2.44) |
| Children | 0.506***(3.84) | 0.140*(1.93) |
| Expenditure | -0.055(-0.40) | -0.024(-0.31) |
| Grandchild care | -0.605**(-2.29) | -0.394**(-2.56) |
| Constant | 15.853***(9.49) | 15.613***(15.75) |
| R-squared | 0.146 | 0.136 |
| F | 110.490*** | 36.850*** |

* p < .1.

** p < .05.

*** p < .01.

**Table S3.** Intermediary effect analysis of urban areas (N = 2,166).

| Mediator effect | β（95%CI） | SE | Z | P |
| --- | --- | --- | --- | --- |
| Financial support |  |  |  |  |
| Indirect effect | -.107***（-.193, -.051） | .035 | -3.01 | 0.003 |
| Direct effect | -.499*（-1.067, .018） | .270 | -1.85 | 0.065 |
| Emotional support |  |  |  |  |
| Indirect effect | -.010（-.062, .037） | .025 | -0.40 | 0.693 |
| Direct effect | -.595**（-1.146, -.062） | .276 | -2.16 | 0.031 |
| Instrumental support |  |  |  |  |
| Indirect effect | -.050*（-.127, -.006） | .030 | -1.69 | 0.091 |
| Direct effect | -.555**（-1.118, -.025） | .271 | -2.05 | 0.040 |

Note:

1. Control variables: Gender, Residence, Education, Marital status, Health, Age, Whether parents live with their children, Number of grandchildren, Number of children, Household per capita consumption expenditure;

2.* p < .1, ** p < .05, *** p < .01.

**Table S4.** A comparative analysis of mediation effect of urban areas based on the KHB method (N = 2,166).

| Mediating variable | β | SE | P Diff | P Reduced |
| --- | --- | --- | --- | --- |
| Financial support | -.098 | .034 | 67.54 | 16.24 |
| Instrumental support | -.047 | .027 | 32.46 | 7.80 |

**Table S5.** Intermediary effect analysis of rural areas (N = 7,053).

| Mediator effect | β（95%CI） | SE | Z | P |
| --- | --- | --- | --- | --- |
| Financial support |  |  |  |  |
| Indirect effect | -.015（-.039, .004） | .011 | -1.41 | 0.157 |
| Direct effect | -.379**（-.713, -.082） | .156 | -2.44 | 0.015 |
| Emotional support |  |  |  |  |
| Indirect effect | -.019**（-.040, -.004） | .009 | -2.07 | 0.038 |
| Direct effect | -.376**（-.672, -.068） | .156 | -2.41 | 0.016 |
| Instrumental support |  |  |  |  |
| Indirect effect | -.057***（-.095, -.033） | .015 | -3.73 | 0.000 |
| Direct effect | -.338**（-.668, -.050） | .156 | -2.17 | 0.030 |

Note:

1. Control variables: Gender, Residence, Education, Marital status, Health, Age, Whether parents live with their children, Number of grandchildren, Number of children, Household per capita consumption expenditure;

2.* p < .1, ** p < .05, *** p < .01.

**Table S6.** A comparative analysis of mediation effect of rural areas based on the KHB method (N = 7,053).

| Mediating variable | β | SE | P Diff | P Reduced |
| --- | --- | --- | --- | --- |
| Emotional support | -.016 | .008 | 22.93 | 4.06 |
| Instrumental support | -.054 | .015 | 77.07 | 13.65 |
